# Supplementary material for: Drug repurposing for aging research using model organisms
Source: Aging Cell. 2017 Jun 16;16(5):1006–15. doi: 10.1111/acel.12626 (PMC5595691; doi:10.1111/acel.12626)
Supplement: Supplementary file 7 — Data S1 Zip‐Archive of all report cards. [file ACEL-16-1006-s007.zip › RC_A1R.pdf]

A1R

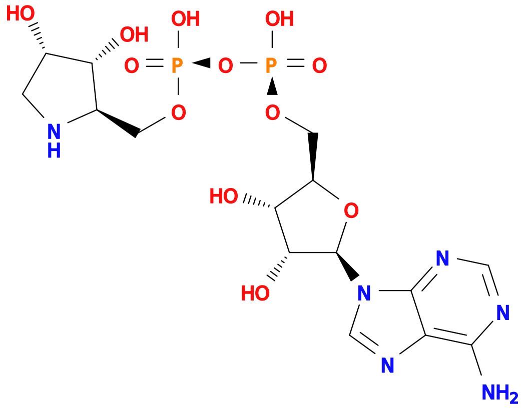

Database identifiers

ChEMBLCompound	CHEMBL1230692
CHEBI	40454

Ranking

	Rank	Score
Drosophila	574/697	0.178
C. elegans	NA	NA

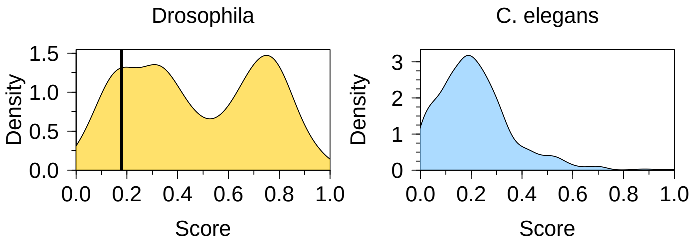

	Ageing implication	Domain conservation	Binding site conservation	Binding affinity	Bioavailability	Lipinski	Promiscuity	Purchasability	Drug approval	**Total**
Drosophila	1.0	0.759	0.694	0.693	(0.9)	-0.15	-0.0	0.0	0.0	0.178
C. elegans	NA	NA	NA	NA	NA	NA	NA	NA	NA	NA

Names

· **[(2R,3R,4S)-3,4-dihydroxypyrrolidin-2-yl]methyl-ADP**
· 5'-o-(s)-(s)-(2r,3r,4s)-3,4-dihydroxypyrrolidin-2-ylmethoxy(hydroxy)phosphoryloxy(hydroxy)phosphoryladenosine
· adenosine 5'-diphosphate (hydroxymethyl)pyrrolidinediol
· ADP-(hydroxymethyl)pyrrolidinediol

Roles

ChEBI entry 40454 has no roles

Status

Approved drug (according to ChEMBL)	No
Number of Rule of 5 violations	3
Binding affinity to original target in log units (RF-Score prediction)	5.81
Burns *C. elegans* bioavailability prediction	-7.24

Compound Target Characteristics

NAD-dependent protein deacetylase HST2

Best gene implication in ageing for this target family came from gene Q9I7I7 annotated in UniProt release 2014_02. Annotation GO 8340 (determination of adult lifespan) was Inferred from Mutant Phenotype

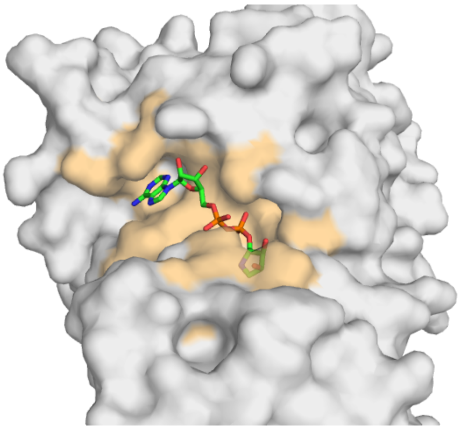

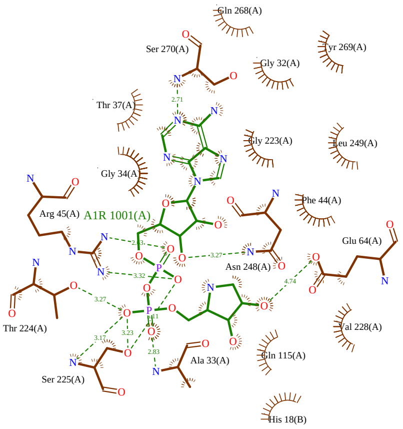

protein	amino acids contacts (binding site)
PDB:2od7:chainA:P53686	G A G T F R E Q G T S V N L Q Y S
PDB:2qqf:chainA:P53686	G A G T F R E Q G T S V N L Q Y S
tr:E9PK80:E9PK80_HUMAN	G A G T F R E Q G T S V N R G D V
sp:Q8IXJ6:SIR2_HUMAN	G A G T F R E Q G T S V N K G E C
sp:Q9NTG7:SIR3_HUMAN	G A G T F R E Q G T S V N R G D V
tr:B2RZ31:B2RZ31_RAT	G A G T F R E Q G T S V N R G D V
tr:C6ZII9:C6ZII9_RAT	G A G T F R E Q G T S V N R G D V
sp:Q5RJQ4:SIR2_RAT	G A G T F R E Q G T S V N K G D C
tr:Q4FJK3:Q4FJK3_MOUSE	G A G T F R E Q G T S V N R G D V
sp:Q8R104:SIR3_MOUSE	G A G T F R E Q G T S V N R G D V
tr:Q3UJK6:Q3UJK6_MOUSE	G A G T F R E Q G T S V N K G D C
sp:Q8VDQ8:SIR2_MOUSE	G A G T F R E Q G T S V N K G D C
sp:Q9I7I7:SIRT2_DROME	G A G T F R T Q G T S V N R G D C
sp:P53686:HST2_YEAST	G A G T F R E Q G T S V N L Q Y S

protein	whole protein ident	simil	domain-based ident	simil	contact-based ident	simil
PDB:2od7:chainA:P53686	0.99	0.99	1.0	1.0	1.0	1.0
PDB:2qqf:chainA:P53686	0.99	0.99	1.0	1.0	1.0	1.0
tr:E9PK80:E9PK80_HUMAN	0.3	0.57	0.41	0.74	0.76	0.72
sp:Q8IXJ6:SIR2_HUMAN	0.3	0.58	0.41	0.7	0.76	0.75
sp:Q9NTG7:SIR3_HUMAN	0.26	0.48	0.38	0.69	0.76	0.72
tr:B2RZ31:B2RZ31_RAT	0.32	0.58	0.4	0.7	0.76	0.72
tr:C6ZII9:C6ZII9_RAT	0.3	0.59	0.4	0.74	0.76	0.72
sp:Q5RJQ4:SIR2_RAT	0.32	0.63	0.4	0.7	0.76	0.73
tr:Q4FJK3:Q4FJK3_MOUSE	0.32	0.58	0.39	0.7	0.76	0.72
sp:Q8R104:SIR3_MOUSE	0.29	0.56	0.39	0.74	0.76	0.72
tr:Q3UJK6:Q3UJK6_MOUSE	0.32	0.63	0.4	0.7	0.76	0.73
sp:Q8VDQ8:SIR2_MOUSE	0.29	0.58	0.4	0.7	0.76	0.73
sp:Q9I7I7:SIRT2_DROME	0.31	0.62	0.4	0.71	0.71	0.69
sp:P53686:HST2_YEAST	1.0	1.0	1.0	1.0	1.0	1.0

Sirt2 (FBgn0038788) associated phenotypes

heat sensitive, lethal - all die before end of pupal stage, some die during pupal stage

(Information from FlyBase)

Sirt2 (UniProt:Q9I7I7) annotation

Function: NAD-dependent protein deacetylase (By similarity). May be involved in the regulation of life span. (, PubMed:17159295).

Cofactor: Zn(2+)Note=Binds 1 zinc ion per subunit. ;

Disruption phenotype: Causes lethality during development. Induced silencing shortens life span. (PubMed:17159295).

(Information from UniProt)
